# Retraction: A Systematic Review and Meta-Analysis of Clinical Effectiveness of Intravenous Magnesium Sulphate in the Treatment of Acute Fast Atrial Fibrillation in Non-surgical Adult Patients

**DOI:** 10.7759/cureus.r257

**Published:** 2026-10-27

**Authors:** Kelvin Mupunga, Willmore Chandavengerwa, Durai Pardon Nyabadza, Adelaide Belinda Masungo

**Affiliations:** 1 Medicine, Northern Ireland Medical and Dental Training Agency (NIMDTA), Belfast, GBR; 2 General Medicine, His Majesty's Correctional Services, Mbabane, SWZ; 3 General Practice, Northern Ireland Medical and Dental Training Agency (NIMDTA), Enniskillen, GBR; 4 Medicine, Omagh Hospital, Omagh, GBR

This article has been retracted by the Editor-in-Chief. Following publication, concerns were raised about the statistical reporting in this article. Post-publication review found errors in the reporting and interpretation of the primary analyses that affect the article’s results and conclusions.

